# Reliability of beat-to-beat blood pressure variability in older adults

**DOI:** 10.21203/rs.3.rs-4190135/v1

**Published:** 2024-04-19

**Authors:** Trevor Lohman, Isabel J. Sible, Fatemah Shenasa, Allison C. Engstrom, Arunima Kapoor, John Paul M. Alitin, Aimee Gaubert, Julian F. Thayer, Farrah Ferrer, Daniel A. Nation

**Affiliations:** University of Southern California, Leonard Davis School of Gerontology; University of Southern California; University of California, Irvine; University of California, Irvine; University of California, Irvine; University of Southern California, Leonard Davis School of Gerontology; University of Southern California, Leonard Davis School of Gerontology; University of California, Irvine; University of Southern California, Leonard Davis School of Gerontology; University of Southern California, Leonard Davis School of Gerontology

**Keywords:** blood pressure variability, beat-to-beat blood pressure variability, continuous blood pressure monitoring, reliability, average real variability

## Abstract

Blood pressure variability (BPV) is emerging as an important risk factor across numerous disease states, including cerebrovascular and neurodegenerative disease in older adults. However, there is no current consensus regarding specific use cases for the numerous available BPV metrics. There is also little published data supporting the ability to reliably measure BPV across metrics in older adults.

BPV metrics were derived from continuous beat-to-beat blood pressure monitoring data. Two sequential 7-minute waveforms were analyzed. Absolute and relative reliability testing was performed. Differences between antihypertensive medication users and non-users on BPV metric reliability was also assessed.

All sequence and dispersion based BPV metrics displayed good test-retest reliability. A measure of BP instability displayed only moderate reliability. Systolic and diastolic average real variability displayed the highest levels of reliability at ICC= .87 and .82 respectively. Additionally, systolic average real variability was the most reliable metric in both the antihypertensive use group, and the no antihypertensive use group.

Beat-to-beat dispersion and sequence-based metrics of BPV can be reliably obtained from older adults using noninvasive continuous blood pressure monitoring. Average real variability may be the most reliable and specific beat-to-beat blood pressure variability metric due to its decreased susceptibility to outliers and low frequency blood pressure oscillations.

## Introduction

Blood pressure variability (BPV) has emerged as a hemodynamic marker of interest with widespread clinical and research utility. BPV is associated with increased mortality (1), and is a risk factor for cardiovascular disease (2-9), stroke (10-12), cerebral small vessel disease (CSVD) (13, 14), neurodegenerative disease (15) and dementia (16, 17), independent of average blood pressure (BP). The regulatory mechanisms which underlie BPV modulation are complex and multifactorial, potentially involving arterial baroreflex sensitivity (18), arterial stiffness (19), endothelial dysfunction (20), and kidney function (20) among other factors (21, 22). While BPV is a clinically relevant hemodynamic marker, little is known about its reliability.

Methodologies for BPV calculation fall into four main categories including very short-term (beat-to-beat) BPV, short-term BPV (<24 hours), medium-term BPV (day-to-day), and long-term BPV (visit-to-visit over months or years) (2). Each category offers unique advantages and disadvantages. Long-term and medium-term BPV are influenced primarily by environmental and behavioral factors such as season, altitude, and antihypertensive medication adherence (23-25). Short-term BPV is reflective of circadian BP rhythms like nocturnal BP dipping and morning BP surge (25, 26) and is influenced by central and peripheral autonomic modulation and arterial elasticity (24,27-29). Very short-term beat-to-beat BPV dynamics yield detailed insights into autonomic and cardiovascular function (23, 30, 31), but have previously required invasive arterial catheterization. The recent validation of noninvasive continuous arterial BP measurement technology (32-34) now enables an accessible and noninvasive methodology for generating validated continuous BP waveforms without arterial catheterization. Numerous hemodynamic markers of interest can be derived from the raw arterial pressure waveforms generated, including standard deviation (SD), coefficient of variation (CV), variability independent of the mean (VIM), and average real variability (ARV). The availability of MRI-compatible devices allows for the study of beat-to-beat BP dynamics during neuroimaging, which may be relevant to cerebrovascular and neurodegenerative conditions associated with increased BPV (35-38).

Numerous studies have identified relationships between BPV metrics and clinically relevant markers of pathophysiology, but no systematic investigation has yet been performed assessing reliability and delineating specific use cases for each metric. Reporting of specific BPV metrics is therefore largely driven by outcomes or convenience. A first step towards elucidating the specific utility and rationale for using a particular BPV metric is to establish its test-retest reliability. However, despite the demonstrated utility of noninvasive continuous BP monitoring, little is known about the reliability of beat-to-beat BPV metrics. The ability to measure BPV accurately and reliably has also been questioned by some (39). To our knowledge, only one study has described BPV test-retest reliability metrics, however the time between tests was 5 years, a time frame over which significant changes in BPV would be expected (30).

The present study addresses this knowledge gap by examining the intrasession test-retest reliability of beat-to-beat BPV metrics in a sample of community dwelling older adults recruited as part of the Vascular Senescence and Cognition (VaSC) cohort. As part of the VaSC study, beat-to-beat BPV was monitoring during brain MRI using an MRI-compatible noninvasive continuous BP device. These data are leveraged in the present study to examine reliability of BPV metrics, including beat-to-beat systolic and diastolic SD, CV, VIM, and ARV, as well as DSBP (maximum SBP minus minimum SBP). The test-retest reliability of noninvasive continuous average systolic BP (SBP), diastolic BP (DBP), and heart rate (HR) are also assessed.

## Methods

### Participants

Participants were recruited from Los Angeles County and Orange County communities, and all procedures were conducted as part of the VaSC Study at the University of Southern California (USC) and University of California Irvine (UCI). Older adults aged 55 to 89 years who were living independently were included. Exclusion criteria were history of clinical stroke, dementia, major neurological or psychiatric disorder or medications impairing the central nervous system, current organ failure or other uncontrolled systemic illness, or contraindication for brain MRI. Study inclusions and exclusions were verified by a structured clinical health interview and review of current medications with the participant and, when available, an informed study partner. This study was approved by the USC and UCI Institutional Review Boards, all participants gave informed consent, and the study was performed in accordance with all relevant guidelines and regulations. The data that support the findings of this study are available upon reasonable request from the corresponding author, DN.

### Continuous BP Data Processing and Analysis

Participants were asked to take medications as normally prescribed and abstain from caffeine the morning of data collection. Beat-to-beat BP measurements were obtained continuously during supine rest in a 3T Siemens MRI scanner, using an MRI compatible non-invasive continuous BP finger cuff device (Biopac^®^). First, the participant rests for 3 minutes in the supine position prior to the calibration period. During calibration, BP waveforms are acquired by the continuous monitoring device and 2 static pressures are simultaneously acquired using a calibrated, MRI compatible automatic BP device with an inflatable brachial artery cuff (TeslaDUO). These static pressures are used to calibrate the continuous BP monitor using the Caretaker^®^ system (Biopac^®^). After calibration, continuous BP was monitored during 2 sequential, 7-minute MRI scans.

**Figure 1:**
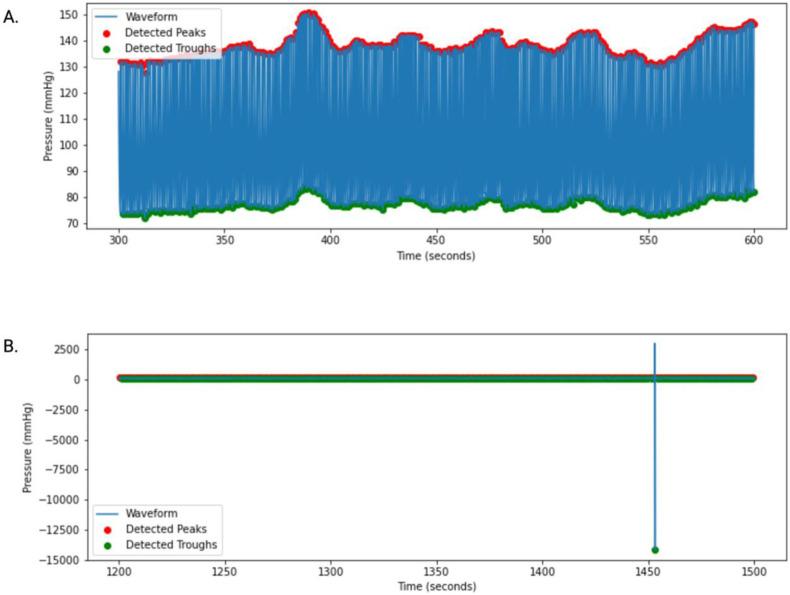
**A:** 300-second continuous blood pressure (BP) waveform with accurate peak (systolic blood pressure) and trough (diastolic blood pressure) detection free from obvious motion artifacts. **B:** 300-second continuous BP waveform with motion artifact visible at 1453.5 seconds.

The Calib upsample utility (Biopac^®^) was used to extract continuous arterial pressure data obtained during the 2 sequential, 7-minute MRI scans at a sample rate of 100 BP readings per second. Data segments free from obvious motion artifacts were selected from each 7-minute continuous BP data segment for further processing. Waveforms were excluded if more than 10% of the data needed to be excluded to remove obvious motion artifacts. Two example 300-second waveform segments, one with an obvious motion artifact and one without, are shown in Figure 1 for illustration purposes.

**Figure 2:**
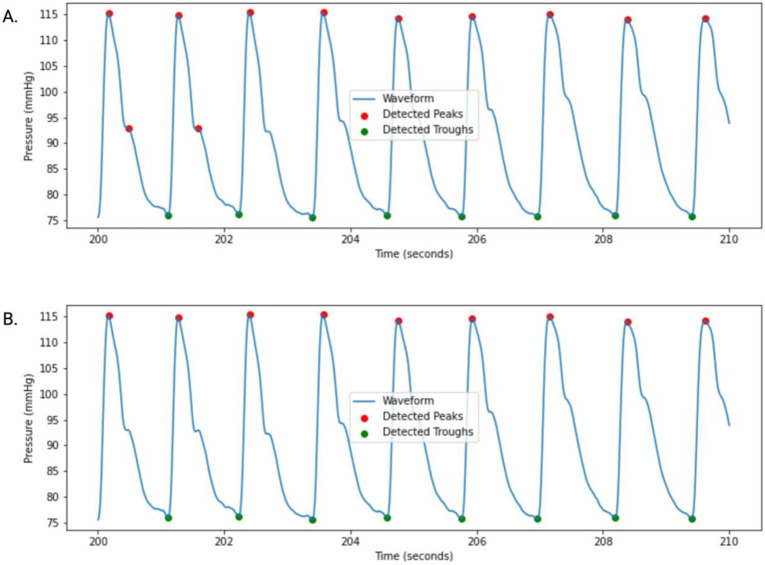
**A:** A 10-second continuous blood pressure waveform with systolic peak and diastolic trough detection applied. Two pronounced dicrotic notches are erroneously detected as peaks by the peak detection algorithm at the 200.5 and 201.6 second marks when using the default minimum detection height and distance parameters (80 mmHg and 40 milliseconds). **B:** The same waveform displayed in Figure 1A is now displayed with a modified minimum distance peak detection parameter of 45 milliseconds. This modification results in accurate peak detection across the waveform.

A peak detection algorithm was used to identify SBP peaks which served as the basis for further cardiovascular parameter calculation. Peaks were detected using the find_peaks function from the scipy.signal library (40), with default parameters set to a minimum detection height of 80 mmHg, and a minimum peak separation of 40 milliseconds by default. Diastolic troughs were identified as the lowest BP reading between two systolic peaks. Each waveform was then visually inspected using the VaSC BP Signal Toolbox application for erroneous or missing peaks and troughs by TL. Occasionally, default data filtering parameters were adjusted as needed to ensure accurate peak detection. A visual illustration of this process is shown in Figure 2. The VaSC BP Signal Toolbox can be accessed at https://github.com/BPLSignal-Toolbox.git after requesting repository access from the corresponding author, DN.

### Calculation of Blood Pressure Variability Metrics

In addition to measurement time, BPV metrics can also be categorized by index type (frequency, dispersion, sequence, or instability) (41, 42). Three measures of BP dispersion (SD, CV, and VIM), 1 measure of BP instability (DSBP), and 1 measure of BP sequence (ARV) were calculated for test-retest comparison in the present study.

**Figure 3:**
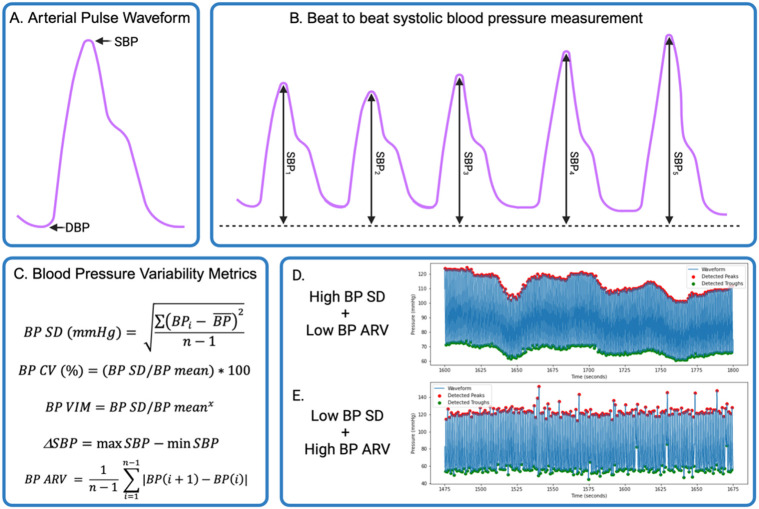
**A:** A single arterial pulse waveform with systolic blood pressure (SBP) peak and diastolic blood pressure (DBP) trough indicated, **B:** Visual representation of continuous blood pressure (BP) waveform with 5 arterial pulse cycles. Systolic peak amplitude is shown to illustrate variation in beat-to-beat SBP. **C:** Five blood pressure variability metric formulas used in the present analysis: SBP standard deviation, coefficient of variation, variability independent of the mean, ∆SBP, and average real variability (ARV). **D:** An example waveform from an individual with high measures of BP dispersion but low measure of ARV. **E:** An example waveform from an individual with high BP ARV but low BP dispersion measures.

The standard deviation of SBP and DBP amplitude measurements (Figure 3A) was obtained across the waveform’s duration as shown in Figure 3B and 3C. BP SD was then further processed into CV and VIM (3). Similar processes were repeated for diastolic BP metrics. Of these metrics, BP SD is used most often due to its straightforward calculation and interpretation, however, it may be correlated with sample mean BP (25, 43). BP CV and BP VIM measures may compliment BP SD because they are independent of mean BP (11, 24, 39,44), allowing for comparison of samples with different means in the case of CV (45) without average BP adjustment. BP CV is calculated as (BP SD/BP mean)*100 (Figure 3C), while BP VIM is calculated by taking BP SD readings divided by mean BP raised to the power of x, where where x was derived from a non-linear fitting of BP standard deviation (SD) against average BP using the nls package in R (Figure 3C). This is then multiplied by the sample mean BP raised to the power of x and rescaled as needed. Nonlinear curve fitting was performed using the nls function in base R (46).

The difference between the maximum SBP reading and minimum SBP reading (DSBP) was included as a measure of BP instability. DSBP is the difference between the maximum and minimum systolic BP readings in a specified window, 7-minutes for the present study.

Systolic and diastolic ARV measures were calculated by taking the absolute differences between consecutive peaks and troughs respectively, and then averaging them across the 7-minute continuous BP waveform (25,47) (Figure 3C). To further confirm the reliability of the continuous BP monitoring methodology the intrasession test-retest reliability of HR and BP were also assessed by comparing the HR, mean SBP and DBP across each selected 7-minute waveform.

### Data Analysis

All statistical analyses were carried out using R (46). Paired t-tests were used to compare mean values of waveform 1 and 2 (test – re-test). Intraclass correlation coefficient (ICC) with a 95% confidence interval was used to assess relative reliability using Munro’s criteria (48) for interpretation while absolute reliability was assessed using the standard error of measurement (SEM), SEM%, smallest real difference (SRD), and SRD%. SEM is calculated as the SD of differences between paired measurements divided by the square root of the sample size (49, 50) while SRD represents the smallest change in a measurement that likely represents a true change rather than a measurement error (49, 51). ICC, SEM, and SRD are commonly used measures of test-retest reliability and are specifically used for this purpose in literature (52-55).

## Results

121 participant visits with continuous BP monitoring were available. Of these, 10 visits were excluded due to poor data quality and excessive motion artifacts. After exclusion, 111 participant visits were included for analysis. Participant characteristics and demographics for this sample are displayed in Table 1. The correlation between all BPV metrics and average blood pressure with and without demographic adjustment, as well as the correlation between all BPV metrics and age is shown in Supplementary File 1.

### Blood Pressure Variability Intrasession Test-retest Reliability

**Figure 4:**
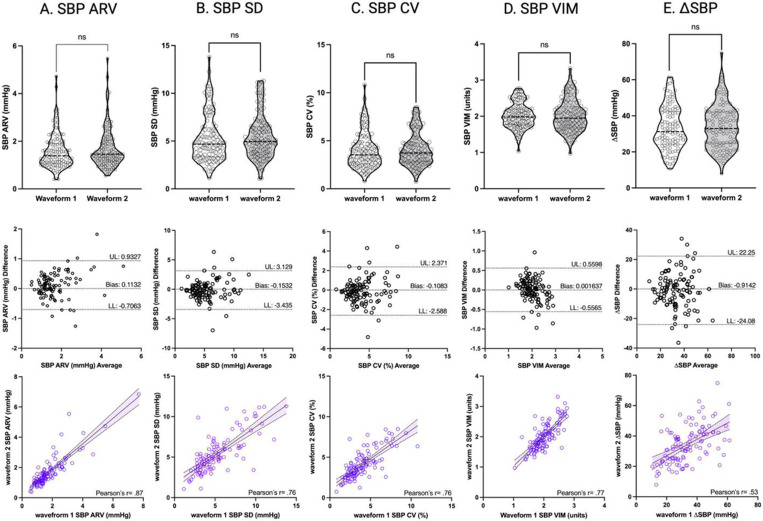
Test-retest violin plots with paired t-test comparison (row 1), test-retest correlation plots with Pearson’s r (row 2) and Bland-Altman plots with bias and 95% limits of agreement indicated by horizontal dashed lines shown for four measures of systolic blood pressure variability: **A)** Systolic blood pressure average real variability, **B)** Systolic blood pressure standard deviation, **C)** Systolic blood pressure coefficient of variation, **D)** Systolic blood pressure variability independent of the mean, and **E)** maximum minus minimum systolic blood pressure.

Table 2 shows the sample mean for each analyzed cardiovascular parameter as well as the paired t-test p-value associated with each test-retest comparison. No significant differences between waveform 1 (test) and waveform 2 (re-test) were observed for any measure. Violin plots display waveform 1 and waveform 2 data distributions for selected measures in Figure 4.

All noninvasive BP waveform-derived cardiovascular parameters displayed excellent absolute test-retest reliability (SEM<5%, SRD<10%). HR, SBP and DBP displayed excellent relative test-retest reliability (ICC > .90), all dispersion (SD, CV, VIM) and sequence (ARV) BPV metrics displayed good relative test-retest reliability (ICC= .70-.89), while DSBP displayed moderate relative test-retest reliability (ICC= .50-.69). These results are shown in Table 3. Correlation and Bland-Altman (56) plots are displayed in Figure 4.

### Antihypertensive treatment and BPV test-retest reliability

SBP ARV displayed excellent test-retest reliability in the group taking no antihypertensive medications, and good test-retest reliability in the group taking antihypertensive medications. All systolic BPV dispersion measures displayed good reliability in the no hypertensives group, but only moderate reliability in the antihypertensive group. Similar observations were seen for the diastolic BPV metrics, except for DBP VIM, which showed improved reliability in the antihypertensive group. Results displayed in Table 4.

## Discussion

The present study finds that sequence and dispersion-based measures of beat-to-beat systolic and diastolic BPV can be reliably derived from a continuous BP monitoring device. Sequence-based metrics, including systolic and diastolic ARV, displayed the highest test-retest reliability in the overall sample. This is likely due to their decreased susceptibility to outliers and low-frequency oscillations in beat-to-beat BP compared to dispersion and instability BPV metrics (57-59). This concept is illustrated in Figure 3D-E, where we can see BP dispersion metrics, such as SD, are more heavily influenced by low-frequency oscillations in BP while BP ARV is more directly influenced by beat-to-beat changes in blood pressure. Low-frequency oscillations may be modulated in part by changes to peripheral vascular resistance (60), transient oscillatory responses to hemodynamic perturbations (61), and intrinsic vasomotor rhythmicity (60) while beat-to-beat changes in BP are mediated by central sympathetic drive, arterial and cardiopulmonary reflexes, and arterial stiffness (25, 62). Additionally, ARV considers the temporal order of BP measurements, adding a time series variability component to the measurement (63) since it reflects the variation in successive differences in beat-to-beat BP. These features of ARV potentially add prognostic value (64) and overcome some potential pitfalls of the SD-based measures which only measure dispersion around mean BP and may be more influenced by outliers (41), while also ignoring the temporal order of BP measurements (2, 47, 64-66). Additionally, two individuals with different BP profiles may have similar BPV dispersion measures but different ARVs, likely making ARV a more specific measure of BPV (67). This may also explain the finding that ARV is a better predictor of 24-hour BPV and subclinical organ damage compared to dispersion metrics (64). Lastly, the measurement of consecutive beat-to-beat differences rather than dispersion from the mean may make ARV particularly well-suited for assessing beat-to-beat variation in BP (2). A parallel can be drawn here to a heart rate variability (HRV) metric, root mean square of successive differences (RMSSD) (68), which acts as a high pass filter thus reflecting the high frequency variability in heart rate and is calculated similarly to ARV (68). RMSSD has been shown to offer certain advantages over other HRV metrics, and it’s possible that ARV may share some of these same advantages such as shorter required sampling durations for reliable measurements (69), and unique insights into parasympathetic tone (70), but further research is needed.

Conversely, DSBP a measure of systolic BP instability, displayed the lowest test-retest reliability, likely due to increased susceptibility to outliers and swings in BP over time (57). All six systolic and diastolic BP dispersion metrics displayed good test-retest reliability, which supports the use of metrics uncorrelated with mean BP like the CV and VIM when comparing groups with different mean BP values. The similar reliability between dispersion measures means that metric choice should be based on context and the individual characteristics of each measure. For example, BP VIM is uncorrelated with the mean and may be useful in research contexts. It is however a scaled, unitless, statistically derived metric that has limited practical use for individuals since it requires that regression coefficients first be derived from a given population (45). Conversely, CV could be used on an individual level and is similarly uncorrelated with mean BP CV is independent of the measurement unit however, so in situations where variability is desired in the original units, BP SD could be used.

When stratified by antihypertensive medication use, SBP ARV displayed excellent reliability in the group not using antihypertensive medications and was the only systolic BPV metric that displayed good reliability in the group using antihypertensive medications. All other measures of systolic BPV displayed moderate or poor test-retest reliability. This is a clinically relevant finding given the widespread use of antihypertensive medication in older adults (71), and supports the use of SBP ARV regardless of antihypertensive treatment status.

The mechanisms responsible for increased systolic BPV are more clearly understood than those associated with increased diastolic BPV. For example, visit-to-visit systolic BPV has been shown to correlate with arterial stiffness (72) and worsening renal function (73), while visit-to-visit DBP variability has not. A key difference between visit-to-visit BPV and beat-to-beat BPV is that visit-to-visit BPV could be influenced by antihypertensive medication adherence (74). For this reason, beat-to-beat BPV may be a more accurate assessment of the underlying physiology which modulates BPV. Regarding this underlying physiology, although not fully understood (41), most studies place central importance on the central sympathetic drive (23, 25), neuronal reflexes (21, 23, 75, 76), and arterial stiffness (72, 77, 78).

The variety of available BPV metrics and relative lack of understanding pertaining to causal mechanisms has resulted in metric choice being largely based on convenience up to this point, and most studies have used less granular and likely less reliable visit-to-visit measures. More investigations should be conducted to differentiate the underlying mechanisms that modulate beat-to-beat BPV metrics and continued assessment of reliability is needed.

The present study supports the use of continuous noninvasive BP derived metrics of BPV in older adults. Of those metrics tested, ARV displayed the highest level of test-retest reliability, perhaps due to its decreased susceptibility to outliers and low frequency oscillations in BP. While these low frequency oscillations reduced the reliability of beat-to-beat dispersion BPV measures, they may not be entirely extraneous, and should therefore continue also to be studied to fully capture the multi-dimensional nature of BPV. All BPV metrics displayed good or excellent test-retest reliability in the present investigation, except for maximum minus minimum systolic BP. Future studies investigating the effects of beat-to-beat BPV should include ARV due to its increased reliability regardless of antihypertensive treatment status, and sensitivity to consecutive beat-to-beat differences in BP.

## Figures and Tables

**Table 1:** Participant characteristics and demographics

Variable Name	Mean (SD) or n (%)
Age (years)	69.89 (6.92) range 55-89
Female (%)	69 (62.2)
VRFs^3^ 2	47 (42.3)
Hypertension	38 (34.2)
High cholesterol	53 (47.7)
Diabetes	11 (9.9)
History of smoking	34 (30.6)
History of cardiovascular disease	11 (9.9)
History of atrial fibrilation	5 (4.5)
History of TIA	2 (1.8)
Using antihypertensive medications	39 (35.1)

**Table 2:** Cardiovascular parameters derived from two sequential 7-minute continuous blood pressure waveforms.

Variable Name	7-minute BP waveform 1 mean±SD	7-minute BP waveform 2 mean±SD	*P*
Heart rate	60.65±10.58	60.19±10.04	.25
Systolic blood pressure	132.65±16.90	131.91±20.75	.39
SBP SD	5.28±2.50	5.43±2.30	.34
SBP CV	4.01±1.88	4.11±1.74	.37
SBP VIM	2.02±.44	2.02±.35	.95
SBP ARV	1.69±.99	1.77±1.02	.10
DSBP	33.32±12.15	34.07±12.23	.42
Diastolic blood pressure	77.42±10.36	77.55±10.50	.44
DBP SD	2.93±1.97	3.08±2.15	.20
DBP CV	3.92±2.93	4.14±3.35	.20
DBP VIM	2.30±1.26	2.30±1.77	.96
DBP ARV	1.90±1.39	1.99±1.35	.20

SBP: systolic blood pressure, SD: standard deviation, CV: coefficient of variation, VIM: variability independent of the mean, ARV: average real variability, DBP: diastolic blood pressure. P-values obtained from paired t-tests.

**Table 3:** Test-retest reliability of cardiovascular parameters obtained from continuous noninvasive blood pressure monitor.

Variable Name	ICC (95% CI)	SEM	SEM%	SRD	SRD%
Heart rate	.98 (.95-1.00)	.19	.31	.27	.43
Systolic blood pressure	.96 (.92-.995)	.48	.36	.67	.50
SBP SD	.76 (.67-.83)	.16	2.98	.23	4.21
SBP CV	.76 (.67-.83)	.12	2.97	.17	4.20
SBP VIM	.75 (.65-.82)	.03	1.34	.04	1.90
SBP ARV	.87 (.82-.91)	.05	2.82	.07	3.99
DSBP	.53 (.38-.65)	1.13	3.35	1.59	4.74
Diastolic blood pressure	.97 (.94-1.00)	.24	.31	.34	.43
DBP SD	.77 (.69-.84)	.13	4.42	.19	6.25
DBP CV	.79 (.71-.85)	.19	4.78	.27	6.76
DBP VIM	.73 (.63-.81)	.11	4.71	.15	6.67
DBP ARV	.82 (.75-.87)	.08	4.06	.11	5.75

ICC: intraclass correlation coefficient, SEM:, SRD: smallest real difference, SBP: systolic blood pressure, SD: standard deviation, CV: coefficient of variation, VIM: variability independent of the mean, ARV: average real variability, DBP: diastolic blood pressure. N=111

**Table 4:** Test-retest reliability of blood pressure variability metrics obtained from continuous noninvasive blood pressure monitor stratified by antihypertensive medication use.

	No antihypertensives n=60	Antihypertensives n=39
Variable Name	ICC (95% CI)	ICC (95% CI)
Systolic blood pressure	.63 (.45-.76)	.98 (.95-.99)
SBP SD	.77 (.64-.86)	.63 (.40-.79)
SBP CV	.75 (.61-.84)	.63 (.39-.78)
SBP VIM	.75 (.61-.84)	.64 (.41-.80)
SBP ARV	.93 (.88-.96)	.81 (.67-.90)
DSBP	.53 (.31-.69)	.49 (.21-.70)
Diastolic blood pressure	.97 (.96-.98)	.98 (.96-.99)
DBP SD	.83 (.73-.90)	.66 (.44-.81)
DBP CV	.86 (.77-.91)	.68 (.46-.82)
DBP VIM	.70 (.54-.81)	.83 (.70-.91)
DBP ARV	.90 (.83-.94)	.70 (.50-.83)

ICC: intraclass correlation coefficient, SBP: systolic blood pressure, SD: standard deviation, CV: coefficient of variation, VIM: variability independent of the mean, ARV: average real variability, DBP: diastolic blood pressure. N=99
